# Whose burden, whose benefit? Revisiting ethical trade-offs in the WHO guidelines on scaling up mass azithromycin administration

**DOI:** 10.1371/journal.pmed.1004736

**Published:** 2025-09-30

**Authors:** Maple Goh, A. M. Viens, Safura Abdool Karim, Aaron S. Kesselheim, Kevin Outterson

**Affiliations:** 1 Program on Regulation, Therapeutics and Law, Division of Pharmacoepidemiology and Pharmacoeconomics, Department of Medicine, Brigham and Women’s Hospital/Harvard Medical School, Boston, Massachusetts, United States of America; 2 Boston University School of Law, Boston, Massachusetts, United States of America; 3 Tufts University School of Medicine, Boston, Massachusetts, United States of America; 4 School of Global Health, York University, Toronto, Ontario, Canada; 5 Global Strategy Lab, York University, Toronto, Ontario, Canada; 6 Berman Institute of Bioethics, Johns Hopkins University, Baltimore, Maryland, United States of America; 7 Centre for the Programme of Aids Research in South Africa, Durban, South Africa

## Abstract

- New evidence suggests that mass drug administration of azithromycin (MDAA) can significantly reduce childhood mortality in high-burden, low-resource settings, yet the World Health Organization’s (WHO) 2020 guidelines take a cautious approach due to concerns about antimicrobial resistance (AMR).
- While the WHO guidelines cite ethical principles, they insufficiently address key considerations, such as intergenerational justice, equitable burden sharing, and the structural determinants of health that shape infectious disease vulnerability.
- Global AMR policy often prioritizes conservation over access in ways that disproportionately burden low-income countries, despite high-income countries also bearing significant responsibility for the emergence and spread of AMR.
- A balanced ethical framework is needed: one that explicitly integrates contextual values, including justice across generations, historical inequities, and community input under uncertainty.
- Revised WHO guidelines that expand eligibility for MDAA based on context-specific criteria, establish thresholds for mortality and resistance monitoring, and encourage global investment in sustainable health systems and antibiotic access, may better align with the WHO’s own principles on equity, human rights, and social determinants of health in the development of guidelines.

## Abstract 2

This Policy Forum article by Maple Goh and colleagues calls for greater consideration of ethical trade-offs relating to conservation and access of antibiotics in the WHO guidelines for mass drug administration of azithromycin.

## Introduction

Mass drug administration (MDA) is a public health intervention involving the simultaneous, periodic, and systematic distribution of medications to entire populations or targeted groups, irrespective of individual disease status [1]. This approach has been widely and effectively used in the control of neglected tropical diseases, such as trachoma, schistosomiasis, and lymphatic filariasis [2].

New evidence suggests that MDA of azithromycin (MDAA), an antibiotic used for the treatment of several types of bacterial infection, could be a strategy to reduce childhood mortality in some low-income countries (LICs) [1,3–6]. Several randomized controlled trials conducted in sub-Saharan Africa have demonstrated that MDAA results in substantial reductions in deaths from respiratory infections, diarrheal diseases, and malaria-related complications [1,6]. The MORDOR trial, conducted in Niger, Malawi, and Tanzania, found that biannual MDAA in children aged 1–59 months led to a 13·5% reduction in all-cause mortality, with the largest effect observed in Niger (18.1%) [6]. The most significant reductions were seen in deaths associated with infectious diseases in the most deprived areas. Similar benefits were reported in the AVENIR trials in Niger, with a 14% mortality reduction [5]. A meta-analysis of these and other studies yielded a pooled mortality risk ratio of 0·85 [7].

Predictably, the evidence of benefit is not consistent across all age groups or settings. Consistent with the AVENIR trials, the LAKANA trial in Mali, which evaluated the impact of MDAA in infants aged 1–11 months, found no statistically significant reduction in mortality from biannual or quarterly azithromycin administration [5,8]. The AVENIR trial also included a head-to-head comparison of outcomes in children aged 1–11 months (statistically insignificant 6% reduction) versus 1–59 months [5]. The results suggest that survival benefit of MDAA may depend on treating the broader age group, rather than infants alone.

Overall, there is consistent evidence that MDAA can significantly reduce childhood mortality in certain high-mortality, low-resource settings, but its effectiveness varies by context and age group. While azithromycin is inexpensive and relatively easy to administer, the delivery of MDA programs in remote, high-mortality settings requires substantial coordination and logistical resources. Nonetheless, data from the MORDOR trial found that the average cost per dose of MDA-azithromycin was $0.74, with a cost per death averted of $898.47 [9]. Though targeted, individualized azithromycin administration may seem preferable from a conservation standpoint, recent evidence from the NAITRE [10] and CHATON [11] trials indicates that this approach does not confer the same mortality benefits as community-wide MDAA. These findings suggest that MDAA’s effectiveness may rely on treating entire communities simultaneously, consistent with mechanisms observed in other MDA programs.

Given these factors, there are now proposals to scale up and implement MDAA intervention in high-mortality, low-resource settings. While MDAA offers promising and immediate benefits, some have expressed concern about the extent to which MDAA contributes to accelerating antimicrobial resistance (AMR) globally and, in light of this, have raised questions about whether MDAA should be pursued in LICs [4,12–14]. AMR is one of the most pressing challenges of the century, projected to directly cause 39 million deaths between 2025 and 2050, and contribute to as many as 169 million deaths overall during this period [15]. The economic toll is similarly stark: AMR is expected to cost up to $1 trillion per year in healthcare expenses by 2050, and cause $3.4 trillion in annual GDP losses annually by 2030, with low- and middle-income countries (LMICs) disproportionately affected [16].

While global trends show rising resistance across nearly all antibiotic classes, including last-line drugs like carbapenems [17], MDAA primarily raises concerns around macrolide resistance. Several studies show rising macrolide resistance following repeated rounds of MDAA [4,13,18,19]; for example, data from trachoma control programs, where MDAA has been used for decades, reported up to a 4-fold increase in macrolide-resistant *Escherichia coli* following MDAA [20–22]. Follow-up studies from the MORDOR trial have shown that MDAA-associated macrolide resistance may spread to untreated populations, with evidence of persistence up to 3.5 years post-MDA [23]. Despite this, the MORDOR II trial compared mortality outcomes between the first and third years of MDAA in communities that had crossed over from placebo to azithromycin, and found no evidence of effect waning over time [3]. However, significant surveillance gaps remain, with many studies not including pre-intervention baseline samples, and only a minority monitoring for non-macrolide resistance or conducting long-term follow-up beyond 2 years [24]. As a result, long-term implications around resistance remain uncertain.

Current global guidelines on MDAA have sought to balance the ethical and practical challenges of the intervention [25]. The World Health Organization (WHO) made two key recommendations:

1. Strong recommendation (low-quality evidence): The WHO advises against the universal implementation of MDAA for the prevention of childhood mortality.
2. Conditional recommendation (low-quality evidence): The WHO suggests that MDAA may be considered in high-mortality settings in sub-Saharan Africa where:
  - Infant mortality is greater than 60 per 1,000 live births, or under-five mortality is greater than 80 per 1,000 live births.
  - AMR trends, adverse effects, and child mortality rates are closely monitored.
  - Other child survival interventions are concurrently strengthened.

These recommendations reflect a cautious approach, driven in part by a need for further evidence. The Guideline Development Group recommended a review within 2–3 years of their 2020 publication [25], with a public notice and comment process launched in November 2024 [26]. As new data on MDAA’s benefits emerge, revised guidelines could allow greater access, thereby saving more lives. Yet, managing AMR risks alongside these benefits requires more than scientific evidence; it requires ethical guidance [27]. WHO guidance can therefore be updated in ways that draw on ethical approaches to provide a fairer balance between antibiotic access and conservation of antibiotic effectiveness.

## Balancing access and conservation in the WHO guidelines for MDAA

Access and conservation remain two central pillars of AMR policy, in which many of our policy responses involve a careful navigation between the tension of balancing these two pillars. There are good reasons to promote access to antibiotics, particularly in LICs where, according to the WHO [25], “[i]t is unethical that populations in low-resource, high-mortality settings should forego the use of potentially life-saving antibiotics.” However, conservation is essential to protect the effectiveness of antibiotics for as long as possible—usually implemented via stewardship measures—and sometimes the scales in modern AMR policy have tipped towards conservation at the cost of access.

MDA poses a particular challenge to navigating this tension because, by its nature, MDA would require giving priority to access to achieve population-level benefits in mortality. Specifically, MDAA requires giving communities or groups antibiotics for the promotion of public health, irrespective of whether the specific individual requires that medication. In this regard, the implementation of MDAA runs counter to the traditional tenets of conservation. This tension is further heightened when seen from a global perspective, as the benefits of MDAA would be felt predominantly in low-resource settings, while the potential contribution to the acceleration of AMR has a wider impact on the global community, perhaps even in countries that do not benefit from MDAA.

However, resolving this tension is more complex than a binary question of whether access or conservation is more important. AMR policy and decision-making involve balancing several ethical values that inform the trade-off between the short-term and long-term benefits associated with access and conservation, especially in the case of MDAA. The WHO Guidelines themselves recognized this, referring to several ethical principles underpinning their recommendations (Table 1).

**Table 1:** Ethical principles underpinning mass drug administration of azithromycin recommendations.

The aims of MDA recommendations in the WHO Guidelines	Ethical principles underpinning the recommendation
Recommendations are directed at the improvement of improve child survival	Achieving the **greater good**, including pursuing health outcomes to promote well-being
Recommendations seek to improve health equity	**Social justice**, which aims for fair health outcomes that reduce disparities
Recommendations ensure a “balance of benefits and potential harms of the intervention”	**Beneficence** and **harm reduction** meaning probable health benefits should outweigh attendant harms
Recommendations are acceptable to, and reflect the values and preferences of, the target population	**Participatory and procedural justice**, where community consultation and local decision-making should inform design and implementation of an intervention
Recommendations are feasible to implement and monitor given resource use considerations	**Effectiveness**, in which an intervention should have some chance at realizing its goal in order to be ethically justified
Recommendations are inclusive of other moral values, such as the importance of avoiding the neglect of marginalized population and promoting autonomy through obtaining consent and allowing opt-outs	**Autonomy** and **justice**, which underpin an aim to promote respect and dignity

Abbreviations: MDA, mass drug administration; WHO, World Health Organization.

The WHO AWaRe classification is one of many components that tackle this balance through grouping antibiotics into Access, Watch, and Reserve categories to guide stewardship and optimize antibiotic use [28]. Azithromycin is included in the Watch group, which contains broad-spectrum antibiotics with a higher potential for developing resistance [29]. Azithromycin is recommended as first-line treatment for several common infections such as infectious gastroenteritis, chlamydia, and gonorrhea [30]. Scaling up MDAA for childhood mortality prevention must therefore be weighed against its potential to compromise the effectiveness of azithromycin for these first-line treatment indications. The AWaRe framework underscores the challenge faced by the guideline committee and national policymakers: Balancing the immediate mortality benefits of MDAA against the long-term stewardship imperative to preserve efficacy for other critical clinical uses. Integrating AWaRe considerations into MDAA-decision aligns with WHO’s broader AMR strategy but would need to recognize the structural context in which national antibiotic stewardship operates. Many countries face constraints such as variable awareness of AWaRe principles, limited surveillance and stewardship infrastructure, uneven availability and affordability of quality-assured antibiotics, and competing policy interests [31]. Therefore, the ability to operationalize AWaRe principles in the balance of access and conservation requires considerations of health systems capacity-strengthening and effective stakeholder engagement, which also entail their own ethical dimensions [31].

It is commendable that the WHO Guidelines include references to these ethical principles in their MDAA recommendations, recognizing that the decision to implement MDAA is not merely a scientific or technical matter, but a value-laden decision. However, we recognize that even these ethical principles underpinning the recommendations are not exhaustive. With the impending revision of the WHO Guidelines, we suggest expanding the ethical principles that inform MDAA decision-making to include other relevant ethical considerations that need to be weighed and balanced to ensure the trade-off between access and conservation in low-resource settings has a robust ethical justification. Specifically, we suggest values that better recognize the historical and present context in which MDAA is implemented, informed by concepts such as burden sharing and equity.

### Risk distribution and burden sharing

AMR does not arise in a vacuum; wealthier nations have historically used antibiotics for comparatively minor or even inappropriate health concerns, such as viral infections, without accounting for the development of AMR or conserving antibiotic efficacy [12]. In fact, a recent systematic review of 412 studies found that approximately 30% of antibiotic consumption worldwide is inappropriate, with no significant difference between LMICs and high-income countries (HICs) after adjusting for access to care (as measured by physicians per capita) [32]. These concerns about conservation concerning MDAA in low-resource settings signal an inconsistency and ethical double standard in how stewardship measures are implemented to favor the interests of HICs in keeping azithromycin effective for as long as possible for their own uses. HICs have robust, well-funded healthcare systems that, to varying degrees, address underlying determinants of health, which can obviate the need for MDAA or similar interventions. The consequence has been that while MDAA provides a potential solution in LICs, it is viewed as ethically unacceptable by HICs that can feasibly and effectively implement more targeted treatment programs.

A reduction in child mortality can generate broader global benefits, including regional economic growth [33–35] and global health security through reduced disease transmission [36]. These positive spillover effects may indirectly benefit HICs and the global community at large. While the potential consequences of AMR may affect a wider global population, the asymmetry underscores the importance of equitable burden-sharing and of articulating clearer principles for how benefits, as well as risks, are distributed across global health interventions.

Given the context of LICs, and specifically low-resource settings, the mortality benefits from MDAA cannot be realized without substantial health systems strengthening. We suggest that, until targeted azithromycin programs for treatment of existing illness can be feasibly implemented in LICs, it is ethically acceptable to adopt MDAA in some settings. In this regard, revising the WHO guidelines to include equitable burden-sharing and fair risk distribution expectations that give more weight to the greater burden of infection and mortality in LICs, and the historical role of HICs in creating and exacerbating AMR, would align with WHO’s stated approach to considering social determinants and resource allocation into guideline development [37]. Global AMR policy could then expand access and invest in underlying determinants of infection and childhood mortality to help protect antibiotic effectiveness for current and future generations in LICs and HICs [38]. HICs can also support research and development for new therapeutics to replace those lost to resistance, making access sustainable through innovation.

### Intergenerational justice

Intergenerational justice concerns the ethical obligations that are owed between generations—past, present, and future—including the overlapping ways in which their lives, preferences, and interests are interconnected. Reference to intergenerational justice in the context of antibiotic use is typically thought to privilege concerns about antibiotic efficacy, leading to stewardship constraints to protect future generations from increased AMR burdens. For instance, it is unproven, but plausible, that increased antibiotic use could lead to more people dying in future generations from drug-resistant infections than those saved at the present time from MDAA-related mortality reduction.

Limiting concern to future generations alone, however, offers a one-dimensional understanding of this concept, neglecting the interconnected roles and obligations of those living now and those who came before within the ethical deliberation underpinning AMR policy. Decisions about the fairness of measures that promote access or conservation can seek to reflect the distributive and ethical implications of how all generations contribute to and are affected by patterns of use and resistance. As such, a concern for intergenerational justice can also support expanding access to reduce mortality now to allow more children to reach adulthood, which allows them to contribute to society and have children, being members of future generations. In reducing present infectious disease burdens, MDAA can also enhance the educational and economic prospects for the next generation, creating intergenerational benefits. While future generations benefit from conserving antibiotic effectiveness, current generations—especially in LIC contexts—may bear disproportionate costs if access is restricted, which is also unjust.

In seeking to balance or make trade-offs between access and conservation, we suggest that the WHO guidance make particular normatively relevant considerations explicit: (1) Their time preferences (e.g., should we prioritize future benefits over immediate benefits?); (2) discounting rates (e.g., should we assign a lower value to benefits for current generations compared to future generations?); (3) intergenerational obligations (e.g., what obligations do current generations have to overlapping and future generations?); and (4) metrics (e.g., on what basis should we measure whether a future generation will be well-off?), all of which inform how and why they may seek to give priority to access or conservation within their recommendations.

### Equity and structural determinants of health

Although equity does feature in the original WHO Guidelines [25], the structural determinants underlying health inequities are not sufficiently addressed. In LICs, higher burdens of disease are shaped by poverty, poor water and sanitation infrastructure, malnutrition, inadequate healthcare, environmental degradation, and insufficient living conditions. These are caused or perpetuated by a variety of factors, including colonialism, historical inequity, neoliberalism, and capitalism [39]. Addressing root causes is complex and expensive, which is why global health often seeks “quick wins,” especially for tackling the health challenges of the world’s poorest people—hence the consideration of MDAA in LICs. The WHO Guidelines could further incorporate a focus on power dynamics, social hierarchies, resource availability, and environmental factors as a means to integrate systemic and structural equity dimensions of AMR, such as gender [40,41], age [42,43], and One Health [44,45]. This is in line with WHO’s own recommendations on the consideration of differential exposures to physical and social environments, differential community and individual vulnerability, and differential access and benefit to health products and services in guideline development [37]. Moreover, the GRADE Evidence to Decision framework also explicitly requires panels to review equity considerations [46]. Though MDAA may be necessary now, long-term strategies must focus on building systems that render such interventions unnecessary.

### Uncertainty and complexity

Accurately predicting the long-term antibiotic effectiveness and AMR consequences of MDAA is challenging, given the number of drivers involved and their interconnection. This complicates the decision-making process and, in turn, impacts the ethical justifiability of MDAA. As a result, predicting the contribution a particular use of MDAA will have to AMR and the harms that can emanate from that are uncertain. Managing uncertainty in public health is always complex, and there are varying approaches. Often, uncertainty is managed through the precautionary principle, which favors deferring the implementation of an intervention if its harms are uncertain, typically until there is more evidence about the potential harms. In the context of AMR, this typically results in the prioritization of conservation over access as a means of averting the potential of serious harm from widespread resistance until there is more evidence about the sustainability of MDAA.

However, this approach has its own problems [47]. In the context of MDAA specifically, the precautionary principle results in depriving specific low-resource contexts of proven, substantial benefits for averting childhood mortality—an issue that implicates justice and equity. The other end of the spectrum is a premature rollout, leading communities to pretend to be exposed to the selective pressure for resistance without receiving any of the intended gains. To effectively navigate this ethical complexity, WHO Guidelines could, if a precautionary approach is adopted, do so with defined parameters that include clear benchmarks and thresholds for revisiting (and potentially revising) decisions, consistent with the GRADE Evidence to Decision framework emphasis on adaptability to specific contexts [46]. This can be explicitly weighed against the other ethical considerations raised above, including a robust stakeholder engagement process to account for the values and preferences of the target population.

## A call for greater emphasis of ethical considerations in the WHO guidelines for MDAA

Revising the 2020 WHO Guidelines for MDAA is warranted, given both the new empirical evidence demonstrating the benefits of MDAA and the need for a more sophisticated recognition of a range of ethical considerations. This evidence, along with findings suggesting that microbiome disruptions are temporary [14,48,49] and resistance patterns vary across contexts [23,24], challenges earlier assumptions about MDA’s sustainability and supports a reassessment of previous guidelines.

This benefit throws the trade-offs around MDAA into sharp relief; curtailing access in the name of conservation may, in certain contexts, deny vulnerable populations a proven, life-saving intervention. A more nuanced view of stewardship is needed: One that includes the obligation to ensure access and to plan for sustainable transitions.

Countries currently scaling up MDAA for child mortality, including those under the 2024 Abuja Declaration, have formally committed to rigorous AMR and mortality monitoring as a condition of implementation [50]. These commitments align with the 2020 WHO Guidelines. However, while policy intent is clear, evidence from recent program evaluations and systematic reviews shows that routine, long-term, and community-level AMR monitoring remains uneven in practice [24]. These surveillance gaps persist for non-macrolide resistance, spillover effects, and genotyping testing, and lack guidance around numerical thresholds that would trigger review, suspension, or modification [13,24]. Due to the recency of the Abuja declaration, it is likely that more time is needed for the implementation of these surveillance measures before reevaluation. Nevertheless, HICs too bear responsibility for the emergence of AMR due to the overuse of antibiotics for non-life-threatening conditions. Consequently, ethical global AMR policy must ensure that LICs are not disproportionately burdened by stewardship demands while being denied access to interventions proven effective in reducing mortality. Decisions about whether low-resource settings should implement MDAA need to be more explicit about the ethical values at play.

The WHO Handbook for Guideline Development outlines the guideline processes that should integrate equity, human rights, gender, and social determinants of health at all stages, supported by systematic and transparent methods for evidence appraisal [37]. This is operationalized through the GRADE approach for evaluating the certainty of evidence, and the Evidence to Decision framework [46] for translating evidence into recommendations. The Evidence to Decision framework explicitly requires panels to consider equity, acceptability, feasibility, and resource use. Reviewing the 2020 MDAA Guidelines through these methodological lenses helps to clarify the extent to which ethical and contextual considerations were integrated into the decision-making process.

Aligning future WHO guidance with principles outlined in the WHO handbook [37] and the Evidence to Decision framework [46] would ensure that new evidence demonstrating the benefits of MDAA in specific contexts is articulated within a clearer framework incorporating relevant ethical principles highlighted within the historical, economic, and social contexts of countries that might consider implementing MDAA. In addition, revised WHO guidelines could expand the conditional recommendation for MDAA beyond the narrow criteria of high infant or under-five mortality rates to incorporate specific criteria for monitoring mortality benefits and AMR trends, with established thresholds for revisiting or revising MDA policies based on emerging evidence and stakeholder perspectives to manage the uncertainty in evidence on harms. Given the complexity of resistance dynamics, the difficulty of predicting long-term outcomes, and the changing healthcare landscape in many LICs, the WHO Guidelines must prioritize adaptability. Ethical trade-offs between access and conservation are not static, and guidance must embrace context-specific and flexible decision-making. Ultimately, the ethical justifiability of MDAA must be grounded in its capacity to deliver real and lasting health benefits to those whose lives and futures are most directly at stake.

## Abbreviations:

AMR: antimicrobial resistance
AWaRe: Access, Watch, and Reserve
HIC: high-income country
LIC: low-income country
LMIC: low- and middle-income countries
MDA: mass drug administration
MDAA: mass drug administration of azithromycin
WHO: World Health Organization
